# Toll-Like Receptor 2 as a Regulator of Oral Tolerance in the Gastrointestinal Tract

**DOI:** 10.1155/2014/606383

**Published:** 2014-09-17

**Authors:** Matthew C. Tunis, Jean S. Marshall

**Affiliations:** ^1^Department of Microbiology and Immunology, Dalhousie University, 5850 College Street, Halifax, NS, Canada B3H 1X5; ^2^Dalhousie Inflammation Group, Dalhousie University, 5850 College Street, Halifax, NS, Canada B3H 1X5

## Abstract

Food allergy, other adverse immune responses to foods, inflammatory bowel disease, and eosinophilic esophagitis have become increasingly common in the last 30 years. It has been proposed in the “hygiene hypothesis” that dysregulated immune responses to environmental microbial stimuli may modify the balance between tolerance and sensitization in some patients. Of the pattern recognition receptors that respond to microbial signals, toll-like receptors (TLRs) represent the most investigated group. The relationship between allergy and TLR activation is currently at the frontier of immunology research. Although TLR2 is abundant in the mucosal environment, little is known about the complex relationship between bystander TLR2 activation by the commensal microflora and the processing of oral antigens. This review focuses on recent advances in our understanding of the relationship between TLR2 and oral tolerance, with an emphasis on regulatory T cells, eosinophils, B cells, IgA, intestinal regulation, and commensal microbes.

## 1. Introduction

The human intestine is a dynamic environment and host to a myriad of bacteria. It is unclear how these commensals regulate immunologic responses to food antigens, but there is mounting evidence that the microbiological environment of the intestine has a profound influence on oral tolerance [1–5]. In addition to the commensals and pathogens residing in the intestine, food products are often contaminated by a wide array of bacteria and fungi. It is likely that contaminating organisms can shape oral tolerance to foods.

While all microbial pattern recognition receptors (PRRs) are likely to have some relationship to food tolerance and allergen processing, TLR2 may be of unique importance due to its expression by intestinal epithelial cells (IECs) and dendritic cells (DCs) in the intestinal environment. Moreover, a majority of commensal bacteria are Gram-positive and thereby have a high capacity for activation of TLR2 [6, 7].

TLR2 is important in identifying bacterial [8] and fungal wall components [9], but it must first combine as a heterodimer with TLR1 or TLR6. The TLR1/2 heterodimer responds to triacyl lipopeptides, while the TLR2/6 heterodimer responds to diacyl lipopeptides and peptidoglycan [10]. Both heterodimers of TLR2 signal through the MyD88-dependent pathway leading to transcriptional activation of NF-*κ*B [11, 12]. TLR2 is expressed by a wide range of cells relevant to mucosal immunity and tolerance including IECs, DCs, T cells, and B cells [13]. While the activation of these TLR2-mediated inflammatory responses is adaptive in the context of pathogenic infection, we are in the early days of understanding how this axis impacts oral tolerance to foods and commensal bacteria. Regulation of IEC permeability [14, 15] and the enteric nervous system [16] both rely on TLR2. Furthermore, although the mechanisms are controversial, several studies report exacerbation of inflammatory bowel disease in the absence of TLR2 [15, 17]. This suggests a critical role for TLR2 in regulating the intestinal microenvironment and local inflammation.

A definitive role for TLR2 expression and activation in the orchestration of tolerance to food antigens has not been characterised. However, growing evidence points to TLR2 as an important factor directing the immunological balance between tolerance and active immune responses to allergens.

## 2. TLR2 Expression and Relationship to Allergy

TLR2 polymorphisms have been associated with deficits in immune regulation such as inflammatory bowel disease, allergic asthma, and atopic disease [18–21]. Notably, a recent study by Nawijn et al. demonstrated that intranasal TLR2 activation concurrent with aerosolized allergen promoted the expansion of allergen-specific regulatory T cells (Tregs) and accordingly suppressed asthma in mice [22]. This is consistent with an earlier observation that sublingual TLR2 agonist therapy concurrent with allergen exposure can abrogate airway hyperresponsiveness in mice [23]. A multitude of studies demonstrate that TLR2 stimulation with systemic or mucosal administration of synthetic agonists can prevent antigen presenting cells from eliciting a T_H_2-polarized response, thereby reducing IgE antibodies and allergenicity in murine asthma models [24–27]. While it is apparent that the delivery of TLR2 agonists via a mucosal route can protect against airway disease in mice, the impacts of TLR2 activation on the processing and tolerance to foods have yet to be characterized. Interestingly, many common foods such as processed meats, chocolate, yoghurt, and cheese contain TLR2 activators [28].

IECs in both the small and large intestine express TLR2, although the distribution and localisation of TLR2 (luminal versus apical) within the cell may vary [29]. In addition, this receptor is expressed by multiple traditional immune effector cells, as described above, often in both intracellular and extracellular compartments. IECs are bathed in an environment replete with TLR2 agonists, such that these cells are probably calibrated to function amid a constitutive level of activation. Accordingly, in TLR2^−/−^ animals, the IEC tight junctions are compromised [14, 15]. Furthermore, TLR2 stimulation promotes tight junctions [14] that could have implications for food processing and the antigen dosage presented to T cells. Defects in TLR2 are associated with inflammatory bowel disease [15, 17] and inappropriate innate responses to intestinal tissue injury in mice [30]. Conversely, intestinal regulation and Treg levels were unchanged in TLR2^−/−^ mice in a chronic model of inflammatory bowel disease [31]. These divergent results may highlight differential roles of TLR2 in chronic inflammation versus acute inflammatory models such as dextran sodium sulphate (DSS) colitis and the different immune cells and mediators driving these models over their respective timeframes [15, 17].

It stands to reason that, in the absence of TLR2, the resulting leaky epithelium could allow amplified allergen dosing and exacerbated allergic responses. The impact of this increased permeability during the initial antigen tolerizing stage is not known. Interestingly, it has also been suggested that IECs are largely unresponsive to TLR2 stimulation, despite their TLR2 expression [32]. This is believed to be an adaptive mechanism to prevent uncontrolled intestinal inflammation in response to the constant barrage of TLR2 agonists from Gram-positive commensals. Consistent with this, TLR2 expression is higher in IECs of the colon compared to the small intestine where earlier food antigen exposure occurs and oral tolerance is thought to be established.

TLR2 functions both as a heterodimer with TLR1 or TLR6 and also potentially as a homodimer. Pam_3_CSK_4_ is a selective ligand for the TLR1/TLR2 heterodimer, while TLR6/TLR1 responds to other ligands such as FSL-1. The distinct role of TLR6 is of particular interest since it is expressed on only selected cell types in the intestine, while TLR1 is much more widely expressed. In a very recent study [33], cells from the intestinal lymphoid tissues activated with anti-CD3 were shown to be more effectively polarized towards T_H_17 and T_H_1 responses by treatment with FSL-1 than with Pam_3_CSK_4_. Using a DSS model of colitis, the TLR6 deficient animals were shown to be disease resistant. In parallel studies of human tissues, TLR6 expression was found to be correlated with the levels of RORC mRNA in inflamed intestines of IBD patients. These results could suggest a role for TLR6 in IBD therapy and have potential implications for the development of T cell responses in the context of TLR6 activators. Clearly, the roles of TLR signaling in the context of inflammatory intestinal disease are not limited to the TLR2 molecule alone.

## 3. TLR2 and Eosinophil Responses

Eosinophils represent an important aspect of chronic allergic disease, and TLR2 may have a key relationship to eosinophils in the mucosal environment within the context of allergy and gastrointestinal inflammation. In animal studies, TLR2 expression and activation were sufficient to facilitate eosinophil recruitment and tissue eosinophilia of the large intestine in the context of experimental colitis [34]. Similarly, eosinophil recruitment to the large intestine and the subsequent chronic inflammatory responses were TLR2-dependent during parasitic* Schistosoma mansoni* infection in mice [35].

A causal link has not been established, but patients with eosinophilic gastrointestinal diseases experience elevated rates of asthma and allergy with up to 76% of patients testing positive for food allergen skin pricks [36]. IgE class switch recombination and local IgE production are also both significantly higher in patients with eosinophilic esophagitis [37]. By contrast, the mucosal administration of a synthetic TLR2 agonist in the airways reliably reduced eosinophilia of the lungs in murine asthma models [24, 26, 38]. TLR2 stimulation therefore has different outcomes on eosinophil tissue homing depending on the activation site and inflammatory status of the tissue in question. The induction of TLR2-dependent eosinophil homing to the intestine may impact the T_H_2 polarization of antigen responses and ultimately alter allergic inflammation or the ongoing regulation of responses to food and the microflora within this compartment.

## 4. TLR2 and the Enteric Nervous System

The interplay between the nervous system and the immune system can be critical for homeostasis and effective immunity. This is particularly true in the intestine where the enteric nervous system (ENS) modifies intestinal motility and epithelial barrier function. TLR2 has been shown to be expressed on enteric neurons, glia, and smooth muscle cells of the intestinal wall. TLR2^−/−^ mice demonstrated disrupted ENS architecture as well as intestinal dysmotility that could be corrected by the addition of glial cell line-derived neurotrophic factor (GDNF). The increased susceptibility to DSS colitis exhibited by TLR2^−/−^ mice can be abrogated by treatment with GDNF. Notably, wild type mice depleted of intestinal microbiota had similar defects in the ENS and intestinal motility to mice deficient in TLR2 [16]. It is not yet clear whether the substantial impact of TLR2 deficiency on ENS function is direct or via secondary effects on the microbiota. However, TLR2 has been implicated in the response to nerve injury in other tissues via the action of local macrophages [39], confirming the importance of this receptor to neuronal function regardless of microbial influences.

## 5. A Relationship between TLR2, Tregs, Microbes, and Tolerance

Oral tolerance can be defined as antigen-specific humoral and cellular hyporesponsiveness following oral antigen exposure [40, 41]. Tolerance is readily induced in mice and humans following oral treatment with food antigen, and food allergy is often considered to result from a failure of oral tolerance mechanisms. The complex process of oral tolerance is known to involve several different cell subsets within the gut associated lymphoid tissues [42], perhaps most notably the Tregs which are required for the induction and maintenance of tolerance to foods [43–46]. Tregs are therefore positioned to profoundly alter the nature of responses to food antigen. Several studies have directly investigated the impact of TLR2 activation on T cell homing and function. Wang et al. demonstrated that TLR2 and MyD88 are necessary for DCs to imprint T cells with intestinal homing markers α_4_ *β*_7_ and CCR9 [47]. This homing is pertinent in light of evidence that Treg intestinal homing to the lamina propria is essential for the ongoing maintenance of oral tolerance to foods [44]. Importantly, lamina propria DC subsets were shown to have high expression of TLR2 relative to other lymphatic DC populations [48]. However, among lamina propria DCs, the CD103^+^ cells, known to be tolerogenic, had lower TLR2 expression compared to other subpopulations. This suggests that TLR2 expression on DCs may not be necessary to drive Treg differentiation.

The expression of Foxp3, associated with Treg development, is abrogated by TLR2 signaling events within the T cell [49]. Similarly, TLR2 activation with the lipopeptide Pam_3_CSK_4_ can abrogate the suppressive capacity of Tregs and DCs* in vitro* [50–52]. Paradoxically, a systemic administration of Pam_3_CSK_4_ promoted the expansion of adoptively transferred Tregs* in vivo* but mitigated their suppressive activity in mice [50]. It may be that the source of Tregs, natural or inducible, impacts the nature and sensitivity of responses to TLR2 stimulation.

It is difficult to reconcile the data above regarding TLR2 abrogation of Treg function, which may be most relevant to oral tolerance induction, with the observation that TLR2 can support the induction of Tregs in the context of commensal microbes; but there is mounting evidence that TLR2 activation by intestinal commensal bacteria can promote local regulatory responses. Microbiota are important for the appropriate maturation of intestinal immunity and this can complicate the interpretation of experimental studies examining the role of bacterial flora in specific immune responses. However, elegant studies with* Bacteroides fragilis* in mice have shown that Tregs induced by TLR2 activation with the unique bacterial polysaccharide A are necessary for successful intestinal colonization [53, 54]. Similarly, the probiotic* Bifidobacterium infantis* promotes Tregs and regulatory cytokine production in humans and functions via TLR2 [55]. It was also recently demonstrated that probiotic* Bifidobacterium breve* induces regulatory IL-10 secreting Tr1 cells via TLR2 stimulation of CD103^+^ dendritic cells, thereby reducing inflammation in the large intestine [56]. Treatment with* Bifidobacterium* components or the TLR2 activation of mast cells by Pam_3_CSK_4_ has even been reported to suppress IgE-mediated mast cell degranulation* in vitro* and* in vivo* [57].

While recent studies show a clear relationship between some commensals and immunologic tolerance, the antigen-specificity of these Treg responses has not been adequately characterized. Moreover, studies exploring commensal Treg induction and the resulting suppression of inflammation tend to examine responses in the colon and cecum, while little attention has been paid to the relationship between commensals and Tregs in the small intestine. The small intestine is an important site of food tolerance induction, and few studies have addressed the role of commensal colonization on food allergy. We do know that commensal bacteria are required for appropriate levels of Tregs to be established in the MLN, and without them oral tolerance is inadequate as shown by studies in germ-free (GF) mice [58]. Furthermore, it has been proposed that the inability of GF mice to establish oral tolerance may be directly related to the failure of these mice to establish adequate T cell populations in the PPs [59]. Several studies have also shown that GF mice display a more T_H_2-polarized response to oral antigens, resulting in IgE antibody production specific to oral antigen and a failure to be tolerized [60–62]. A recent study by Noval Rivas et al. demonstrated that variations in the murine commensal flora will dictate the balance between oral tolerance and allergy to oral antigen through involvement of Treg populations [5]. The changes in Treg and humoral responses to food antigen in the context of commensals are likely to implicate TLRs, but more directed investigations must be carried out to fully understand the precise role of TLR2 signaling in food tolerance.

## 6. TLR2 Directs B Cell and IgA Responses

IgA is the most abundant mucosal antibody, with an average of 5 g secreted daily in human feces [63]. IgA occurs both as a monomer in serum and as a dimer bound by the J-chain. The IgA dimers are translocated to the gut lumen and to other mucosal surfaces by the poly-Ig receptor (pIgR) on IECs, where the antibodies participate in the immune exclusion of microbes. The relationship between secreted IgA and food allergy has not been fully elucidated, but patients with selective IgA deficiency demonstrate impaired mucosal immunity and deficits of intestinal regulation that correlate with higher rates of food allergy and inflammatory bowel disease [64, 65]. Further to this, secreted IgA has been correlated with improved tolerance to peanut challenge in allergic patients [66]. Both serum and secreted antigen-specific IgA have been shown to prevent oral anaphylaxis [67] and allergic diarrhea [68] in mice, suggesting that IgA responses can be protective in the context of an oral allergen challenge. Elevated secreted IgA has been documented in mice treated with oral food antigen compared to naïve animals [69], and antigen-specific IgA is detected in the serum of mice upon oral immunotherapy treatment [70]. Thus, it appears likely that robust IgA production is related to protection against allergic responses to food. TLR2 stimulation has well-documented effects on B cell activation and local IgA responses.

Both naïve and activated B cells express TLR2 [71]. Therefore, in addition to activating IECs, DCs, and T cells in the mucosal environment, TLR2 ligands can act directly on B cells. It was recently reported that TLR2 activation of resting murine B cells in concert with CD40L stimulation can dramatically enhance proliferation, class switch recombination, and plasma cell differentiation [72, 73]. Work by Jain et al. has also shown that TLR2 activation of B cells enhanced their ability to respond to CD40 stimulation by T cells upon antigen presentation [73]. Consistent with this, Pam_3_CSK_4_ treatment of naïve human peripheral B cells results in production of IL-6 and IL-13 [74], both of which can promote B cell activation and antibody production. Of particular relevance to oral tolerance, TLR2 stimulation of B cells with synthetic lipopeptide resulted in the proliferation of Peyer's patch B cells and subsequent antibody production in a murine model [71]. Furthermore, stimulation of human B cells with TLR2 agonists promotes IgA production, J chain production, and the expression of intestinal homing markers CCR9 and CCR10 [75]. Prior to the characterization of TLR2, an older study with lipopeptides found that oral administration of Pam_3_CSK_4_ (now known to be a TLR2/1 agonist) concurrent with oral antigen promoted significant antigen-specific plasma IgA and secretory fecal IgA responses in a murine model [76]. Finally, expression of the pIgR and transcytosis of IgA dimers across IECs are impaired in the absence of MyD88 signaling [77]. Taken together, these findings identify key roles for TLR2 in regulating B cell maturation, expansion, homing, IgA production, and even IgA secretion.

Evidence points towards B cell activation and IgA production as necessary to contain commensal microbes to the intestinal lumen [78, 79]. The same principle may apply to food antigens, but the antigen-specificity of activated B cells is at issue. Further investigation is necessary to elucidate whether bystander TLR2 activation of intestinal B cells by commensal bacteria or food contaminants is capable of promoting expansion of food-specific B cells and the associated IgA response.

## 7. Summary

**Figure 1:**
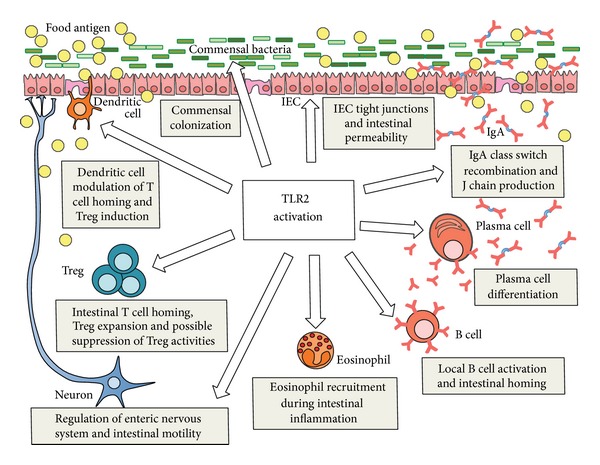
Proposed involvement of TLR2 in oral antigen responses of the intestinal microenvironment. This figure outlines several suggested roles of key intestinal cell types in the regulation of oral tolerance to oral antigens in the context of TLR2 activation.

TLR2 is increasingly at the forefront of intestinal immunology investigations. TLR2 stimulation promotes intestinal barrier function, B cell maturation, mucosal homing, and IgA responses (Figure 1). TLR2 activation by some commensal species facilitates Treg differentiation. However, most reports indicate that the direct impact of TLR2 stimulation on Tregs is to suppress their function once induced, and systemic TLR2 activation promotes intestinal homing of eosinophils during intestinal inflammation and impacts enteric nerve function (Figure 1). It is likely that the TLR2-dependent axis of regulation and allergic sensitization is plastic and responsive to changes in TLR2 agonist dosing. Furthermore, the physiological site of activation may be critically important in dictating subsequent responses. For example, constitutive low grade commensal TLR2 stimulation may support tolerance to foods, but a breach in the mucosal barrier and amplified TLR2 agonist dosing may promote local inflammation and sensitization to bystander food antigens. Such a scenario needs to be tested experimentally in order to better understand the relationship between TLR2 and food allergy. Finally, a dedicated comparison between TLR2 activation in the small and large intestine and the subsequent Treg and B cell responses would be extremely important for understanding the implications of TLR2 in food tolerance and allergy. There is a true deficit of small intestine research in this field.

Unfortunately, the most fundamental question remains unanswered: does TLR2 activation support or disrupt human oral tolerance to food antigens? As described above, a number of lines of evidence suggest that this may be the case, but there is insufficient evidence available to move forward with TLR2 targeted prevention or treatment strategies. With the current interest in host-commensal interactions and the growing importance of food allergy, we are sure to see rapid advancements in this area that will have implications both for allergic disease and for effective oral immunization.
